# Methods to Discover and Evaluate Proteasome Small Molecule Stimulators

**DOI:** 10.3390/molecules24122341

**Published:** 2019-06-25

**Authors:** Rachel A. Coleman, Darci J. Trader

**Affiliations:** Department of Medicinal Chemistry and Molecular Pharmacology, Purdue University, 610 Purdue Mall, West Lafayette, IN 47907, USA; colema84@purdue.edu

**Keywords:** proteasome, stimulation, fluorescent probes, 20S CP

## Abstract

Protein accumulation has been identified as a characteristic of many degenerative conditions, such as neurodegenerative diseases and aging. In most cases, these conditions also present with diminished protein degradation. The ubiquitin-proteasome system (UPS) is responsible for the degradation of the majority of proteins in cells; however, the activity of the proteasome is reduced in these disease states, contributing to the accumulation of toxic protein. It has been hypothesized that proteasome activity, both ubiquitin-dependent and -independent, can be chemically stimulated to reduce the load of protein in diseased cells. Several methods exist to identify and characterize stimulators of proteasome activity. In this review, we detail the ways in which protease activity can be enhanced and analyze the biochemical and cellular methods of identifying stimulators of both the ubiquitin-dependent and -independent proteasome activities.

## 1. Introduction

**Figure 1:**
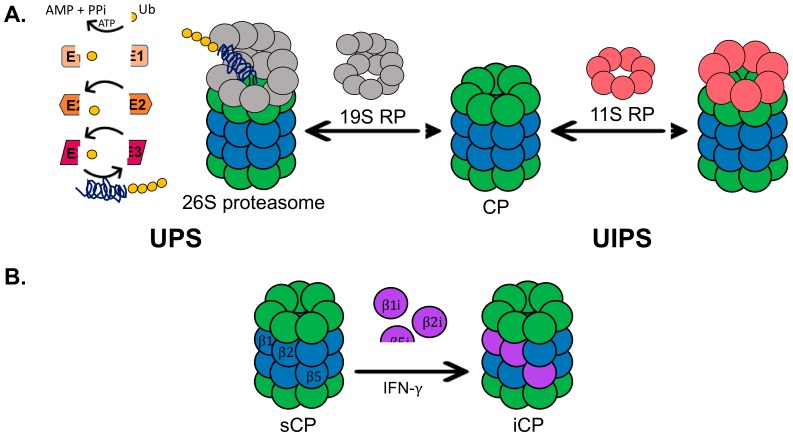
(**A**) Overview of the ubiquitin-proteasome system (UPS) and the ubiquitin-independent proteasome system (UIPS). The UPS utilizes a set of E-ligases to polyubiquitinate protein substrates for recognition and degradation by the 26S proteasome. The UIPS employs the 20S core particles (20S CP), which can be capped or uncapped by the 11S regulatory particle (11S RP), to degrade oxidatively damaged and intrinsically disordered proteins. (**B**) In the presence of pro-inflammatory signals, such as interferon-gamma (IFN-γ), the catalytic subunits of the standard 20S CP (sCP) can become exchanged for the catalytic subunits of the immunoproteasome (iCP).

One of the most basic, essential cellular processes is the degradation of proteins. This primarily occurs through two pathways: degradation by lysosomes, also known as autophagy, or degradation by the proteasome [1,2]. The proteasome degrades proteins using two systems, the ubiquitin-dependent (UPS) and the ubiquitin-independent (UIPS) proteasome systems, Figure 1A [3,4]. The UPS utilizes the 26S proteasome isoform, which is comprised of a 19S regulatory particle (19S RP) and a 20S core particle (20S CP). The 19S RP is responsible for recognizing a ubiquitinated substrate, removing ubiquitin, unfolding the substrate, and shuttling it into the 20S CP. The physical association of the 19S RP to the 20S CP causes a conformational change in the α-ring of the 20S CP to allow the protein substrate to enter for hydrolysis [5]. The 20S CP is composed of four heptameric rings in the order α, β, β, α. The α-subunits form the gate of the 20S CP, which prevents well-folded proteins from entering the hydrolysis chamber of the 20S CP without a regulatory particle present [6]. The β-rings of the 20S CP each house three catalytic subunits, β1, β2, and β5, which correspond to the following hydrolysis activities: caspase-like, trypsin-like, and chymotrypsin-like, respectively. The UPS requires a system of proteins to polyubiquitinate substrates that are recognized and degraded by the 26S proteasome. A well-characterized pathway in which the UPS plays a critical role is the endoplasmic reticulum-associated protein degradation (ERAD) system [7,8]. ERAD focuses on the degradation of misfolded proteins, and many disease states characterized by ER stress are correlated with a decrease in UPS activity [9,10]. The UIPS, on the other hand, cannot degrade ubiquitinated substrates. This system utilizes the 20S CP for the degradation of intrinsically disordered or oxidatively damaged proteins that become unfolded [11,12]. These proteins are capable of entering the gate of the 20S CP without the presence of the 19S RP to initiate a conformational change of the α-ring. However, the UIPS can make use of other regulatory particles, such as the 11S RP, which can cause a similar conformational change of the α-ring to expedite substrate entry [13].

In healthy cells, the UPS and UIPS are both working to help alleviate the load of damaged proteins or proteins that have served their purpose and need to be degraded. In healthy, young fibroblasts, the 26S proteasome isoform only accounts for approximately 21 to 35% of proteasome isoforms in the cell [14]. The remaining percentage is comprised of the 11S RP-capped 20S CP and free 20S CP, which is the primary isoform, accounting for two-thirds of the proteasome population. In immune cells and cells exposed to pro-inflammatory signals, the standard 20S CP can become exchanged for the immunoproteasome, Figure 1B [15]. The immunoproteasome is structurally identical to the standard 20S CP, except for the catalytic subunits, which are exchanged for the β1i, β2i, and β5i [16]. These subunits have different hydrolysis activities than the standard 20S CP, resulting in alternative cleavage sites of protein substrates. The immunoproteasome produces peptides capable of being loaded into MHC-I complexes to alert the immune system of an infection.

Many disease states have been characterized by an accumulation of protein, leading to proteotoxicity. Diseases, such as Alzheimer’s, Parkinson’s, aging, Huntington’s, and amyotrophic lateral sclerosis, exhibit a diminished ability of cells to maintain proper proteostasis [17,18,19,20,21,22,23]. This is due, in part, to reduced activity of the proteasome. In the majority of these diseases, this phenomenon is the result of a lack of expression of subunits composing the 19S RP, increasing the amount of free 20S CP, or both the 19S RP and 20S CP, decreasing the total quantity of proteasomes in the cell [24]. Because this is a shared characteristic of many disease states, developing a method to enhance proteasome activity, either 26S or 20S CP, to reduce protein accumulation has become a prominent area of research. Multiple studies have found that increased proteasome activity is beneficial, decreasing the accumulation of oxidized protein and delaying aging [25,26,27]. Enhancing proteasome activity can be accomplished through genetic manipulation of subunit expression, such as overexpressing the β5 subunit of the 20S CP or Rpn-6 of the 19S RP, or through peptide or small molecule stimulation [26,28,29,30]. Chemical stimulators are commonly grouped into two categories: gate-openers, which modulate the α-ring of the 20S CP to enhance substrate entry, and allosteric modulators of the hydrolysis activity, which can impact at least one of the activities performed by the catalytic β-subunits. Several peptides or small molecule stimulators of the proteasome have been identified by a few different research groups, and the methodology to detect and characterize proteasome stimulators is an evolving field [31,32,33,34,35,36,37,38,39,40,41,42,43]. In this review, we describe and assess various current techniques used to discover and evaluate small molecule stimulators of the proteasome.

## 2. Discovery: Biochemical High-Throughput Screening Assays

**Figure 2:**
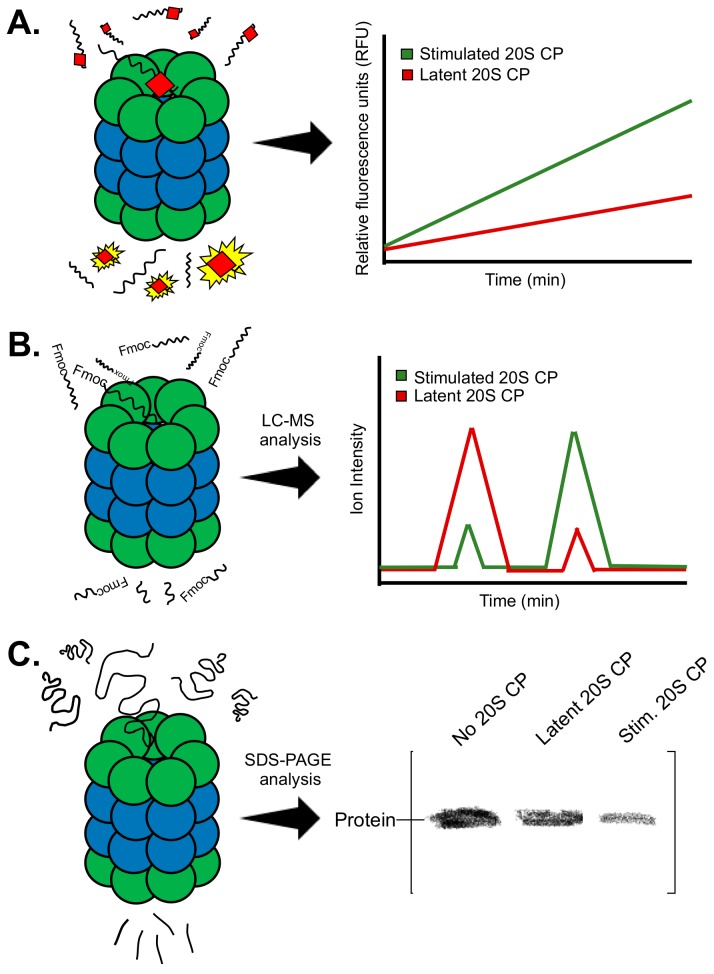
Summary of the biochemical methods often used to identify proteasome stimulators. (**A**) Fluorogenic substrates produce a fluorescent signal following substrate hydrolysis. (**B**) Analysis of the degradation of peptide substrates can be performed using mass spectrometry. The first peak in the spectra corresponds to the full peptide. The second peak corresponds to the degradation product containing the fmoc protecting group. (**C**) Evaluating degradation of a specific protein by the proteasome can be performed using SDS-PAGE analysis.

Initial efforts to discover proteasome stimulators are often performed using high-throughput biochemical screens. These methods use purified proteasome and monitor proteasome activity through co-incubation with a substrate (Figure 2A–C). In this section, we will review the different techniques used to monitor proteasome activity and describe how these methods can also be used to extrapolate a general mechanism of stimulation of a “hit” molecule.

### 2.1. Fluorogenic Peptide Substrates

Since the discovery of the proteasome in 1983, fluorogenic peptide substrates have been used to monitor its activity. In the initial discovery, Wilk and Orlowski used peptides’ three amino acids in length containing either a p-nitroanilide (pNA) or a 2-napthylamide (2NA) on the C-terminus [44]. The peptides used were specific to the three protease activities exhibited by the proteasome. Following the cleavage after the third amino acid residue, the pNA or 2NA group would fluoresce. Monitoring this fluorescence over time allows one to assess proteasome activity. Since 1983, many more fluorogenic peptide substrates have been synthesized for each of the activities of the proteasome [45]. The most commonly used employ a 7-amino-4-methylcoumarin (AMC) group on the C-terminus. This group has a higher degree of fluorescence over pNA or 2NA and therefore, provides a more effective readout [46]. Table 1 lists the fluorogenic peptide substrates commonly used today and the corresponding hydrolysis activity of the proteasome.

**Figure 3:**
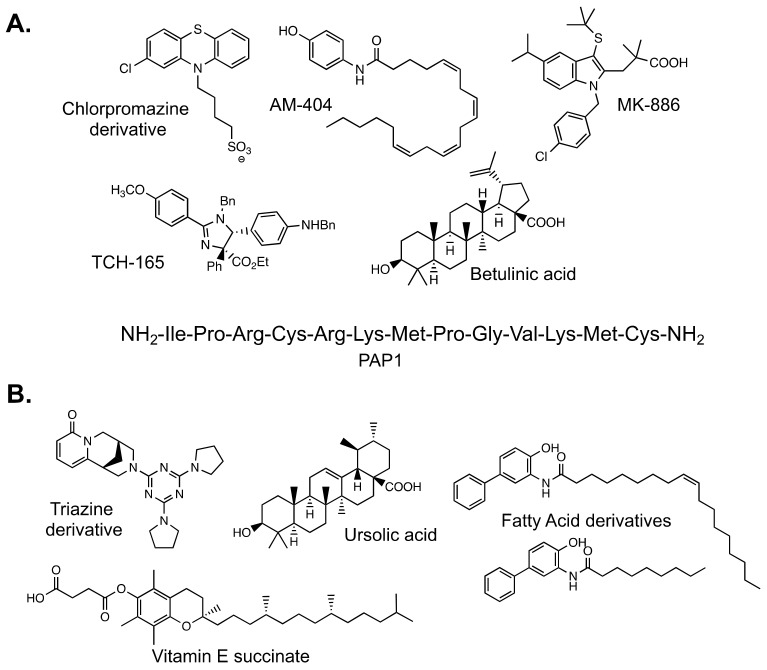
(**A**) Structures of small molecule and peptide stimulators discovered using Suc-LLVY-AMC to monitor proteasome activity. All except betulinic acid are believed to act as gate-openers of the 20S CP. Betulinic acid has been shown to enhance only the chymotrypsin-like activity of the β5 subunit. (**B**) Structures of small molecules determined to be proteasome stimulators using the fluorescence resonance energy transfer (FRET) reporter. All except the triazine derivative are gate-openers of the 20S CP. The triazine derivative enhances a β-subunit catalytic activity, although the precise subunit is unknown.

The majority of these substrates are small (3–4 amino acids in length) and can easily fit past the gate of the 20S CP to be hydrolyzed, allowing for efficient measurement of activity. Multiple studies that have identified proteasome stimulators have utilized Suc-LLVY-AMC [32,34,35,36,38,39,40,41]. By plotting the change in fluorescence of AMC over time, one can obtain a “rate” of hydrolysis. This “rate” is used to compare a sample containing a tested small molecule to a control sample to determine if there is an increase or decrease in activity. Small molecules and peptides discovered as stimulators of the proteasome using this method are shown in Figure 3A.

A more recently developed reporter of proteasome activity utilizes fluorescence resonance energy transfer (FRET) technology to produce a fluorescent signal [33]. This reporter is larger than the commonly used AMC reporters and contains the FRET pair, Edans and Dabcyl. While the peptide is intact, FRET occurs between the pair, producing only a minimal fluorescent signal that does not change over time. However, once the peptide is hydrolyzed, the distance between the FRET pair increases, producing a much greater fluorescent signal. Similar to the AMC reporters, this signal can be monitored over time to assess proteasome activity. This reporter is not specific to any of the hydrolysis activities of the proteasome and was developed to be similar to a protein-like substrate. Due to its size, it does not easily fit into the hydrolysis chamber of the 20S CP. Characterization of this reporter revealed that it is more sensitive to 20S CP stimulation than the Suc-LLVY-AMC substrate. Figure 3B shows the stimulating small molecules discovered using the FRET reporter [33,42].

### 2.2. Mass Spectrometry Substrate Analysis

Another effective method for identifying stimulators of proteasome activity involves the use of mass spectrometry (Figure 2B). First developed by Trader et al., this method employs an N-terminally Fmoc-protected peptide substrate that is incubated with or without the proteasome for a certain period of time [34]. The sample is then quenched and run on a mass spectrometer to identify and quantitate the full peptide and the cleavage product(s). This provides the basal level of activity of the proteasome. If a small molecule is included in the incubation for testing, quantitation of the full peptide and cleavage product(s) from the chromatogram can reveal whether this molecule has any effect on proteasome activity. For this assay, the Fmoc group is kept on the *N*-terminus of the substrate to facilitate retention of the peptide on the reverse-phase column. This allows for the full peptide and the cleavage product containing the *N*-terminus to be easily identified and quantified on the chromatogram. For normalization, results between samples are compared by assessing the ratio of cleaved product to the whole peptide. This value was used to compare samples and determine the extent of increased proteasome activity induced by a small molecule.

### 2.3. Gel-Based Assays

The methods described to this point utilize peptide substrates to assess proteasome activity. Many times, small molecule enhanced degradation of a peptide substrate does not directly translate to enhanced degradation of a protein. Gel-based assays have been developed to monitor protein substrate degradation by the proteasome (Figure 2C) [32,33,34,35]. These assays utilize purified proteins, both proteasome and substrate. The protein substrate is incubated (1) by itself, (2) with the proteasome, and (3) with the proteasome and a small molecule. The samples are then run on an SDS-PAGE gel and can either be stained with Coommassie, or an immunoblot can be performed to visualize the protein substrate. Imaging and subsequent quantitative analysis are then performed to determine how much of the substrate is degraded by the proteasome in the absence and presence of the small molecule. It is important to obtain a standard curve of the protein substrate before performing this assay to indicate the limit of detection of that particular substrate when used for an immunoblot or stained with Coommassie. The amount of proteasome used will also need to be optimized to prevent the presence of the proteasome from interfering with substrate quantitation of a Coommassie-stained gel. This assay is useful to determine how much more of a particular protein can be degraded in the presence of a small molecule stimulator, but it can also be used to assess a rate of degradation if performed for different time periods. Kinetic analysis using this assay can be a very beneficial characterization of a small molecule stimulator of the proteasome.

### 2.4. General Mechanism of Small Molecule Stimulators

All of the assays described above can be used as primary or secondary screening to either identify or validate a small molecule stimulator of the proteasome (any isoform). Because these assays can be used with either the 20S CP, the 26S proteasome, or the immunoproteasome, repeating the assay with the other isoforms can provide an indication of the mechanism of stimulation. In theory, small molecule stimulators can act through two separate mechanisms: (1) gate-openers, which stabilize a more gate-opened state of the 20S CP (or iCP), or (2) an allosteric modulator, impacting at least one of the hydrolysis activities [33]. Gate-opening small molecules should not have any effect on the activity of the 26S proteasome, as the gate is occupied by the 19S RP. These types of stimulators should, however, still maintain a stimulatory effect with the immunoproteasome, which has a gate structurally identical to that of the 20S CP. A molecule that is able to directly impact one of the hydrolysis activities will also have this effect on the 26S proteasome but will likely not affect the immunoproteasome. Because the immunoproteasome contains different catalytic subunits, an allosteric modulator of β1, β2, or β5 of the 20S CP should not impact β1i, β2i, or β5i of the immunoproteasome. However, these subunits maintain a high percentage of structural similarity, so an impact on activity may still be observed [16].

Of the two general mechanisms, allosteric modulators are less common and less understood. There is evidence that some allosteric modulators may have different impacts on the various hydrolysis activities. For instance, Ritonavir, a known human immunodeficiency virus-1 protease inhibitor, was shown to inhibit the chymotrypsin-like activity while enhancing the trypsin-like activity of the proteasome [47,48]. This type of impact could indicate multiple binding sites; however, further structural studies are necessary to understand the mechanism behind this allosteric modulator.

## 3. Discovery: In Vitro Assays

Following the identification and validation of small molecule proteasome stimulators, cellular assays are often performed to determine the efficacy of these stimulators in a cell model. Such assays provide information on cell permeability and target specificity, both of which are important for a potential therapeutic approach and for understanding the overall impacts of stimulating the proteasome. In this section, we review the cellular assays most commonly used to assess proteasome stimulators and discuss assays that have been used to monitor proteasome activity, which could be used to evaluate stimulators.

### 3.1. GFP-Fusion Protein Assay: 20S CP Stimulator

**Figure 4:**
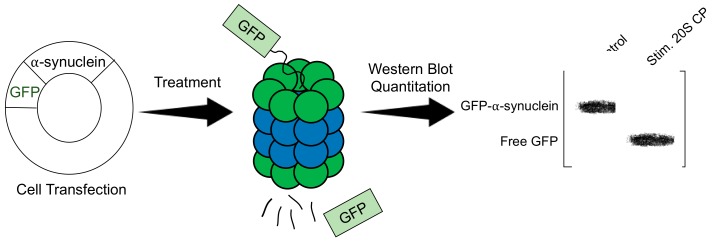
General procedure of evaluating a 20S CP stimulator in cells using a GFP-α-synuclein fusion protein.

Stimulators of the 20S CP are often examined using a green fluorescent protein (GFP)-fusion protein (Figure 4) [32,34,35]. Cell lines suitable for transfection are transfected to express a known substrate of the 20S CP that has been fused to GFP. Common protein substrates include α-synuclein, tau, and ornithine decarboxylase. This assay is effective at monitoring 20S CP-mediated degradation, as the uncapped 20S CP can degrade the unstructured portion of the GFP-protein fusion, leaving an increased amount of free, intact GFP, whereas the 26S proteasome can degrade the entire fusion protein. The transfected cells can be treated with a 20S CP stimulator to be compared to a control sample. The time of treatment is dependent on the toxicity of the molecule; however, most studies using this assay are performed using a 24-hr treatment period. Following treatment, the cells are lysed, and a Western blot is performed, looking specifically at GFP. The analysis is performed by quantitating the bands corresponding to the fusion protein and free GFP. The ratio of free GFP to the fusion is calculated for the treated sample and compared to the control to determine the increased activity of the 20S CP. One of the necessary assumptions of this assay is that the activity of the 26S proteasome is consistent across all samples. Because the 26S proteasome will degrade the entire fusion protein and the free GFP that is produced by 20S CP activity, it is important that the activity of the 26S proteasome is the same across all samples. This assay can effectively be used once it is determined that the small molecule does not impact 26S proteasome activity.

### 3.2. Analysis of Endogenous Protein Levels

Evaluating the impact of a proteasome stimulator on the degradation of an endogenous protein can often be difficult to assess due to ongoing protein synthesis. To more easily detect enhanced degradation, many studies have employed cycloheximide, a protein synthesis inhibitor [49]. Cycloheximide exerts its effects by inhibiting translational elongation in eukaryotes, making it a useful tool to evaluate the degradation of proteins. Often, cycloheximide is used to determine the half-life of a particular protein [50]. This is accomplished by treating cells with cycloheximide for various time periods and performing a Western blot for that protein to see at what point the band disappears. Cycloheximide is the most commonly used agent for these purposes; however, protein synthesis inhibition by cycloheximide is not 100% efficient. The transcription inhibitor α-amanitin is a potent protein synthesis inhibitor and can be used as a substitute [51].

When using cycloheximide, or any protein synthesis inhibitor, to evaluate a proteasome stimulator, the endogenous protein of interest is often one that is already known to be degraded by at least one isoform of the proteasome. For example, α-synuclein is a known substrate of the 20S CP and is commonly used to evaluate the impact of a 20S CP stimulator in neuronal cells [34]. When performing an experiment to look at endogenous protein, it is important to understand which isoforms of the proteasome will be impacted. This can be done using the biochemical assays mentioned above. To analyze stimulators of the 26S proteasome, it can be beneficial to look at the ubiquitinated form of the protein of interest. Substrates of the 26S proteasome are polyubiquitinated for recognition by the 19S RP [52]. Therefore, it can be helpful to analyze the ubiquitinated protein levels when evaluating protein degradation by the 26S proteasome. Analysis of endogenous protein levels can be performed by Western blot.

## 4. Methods to Monitor Proteasome Activity in Cells

### 4.1. GFP-Fusion Protein Assay: 26S Proteasome Stimulator

GFP-fusion proteins have also been specifically designed to monitor 26S proteasome activity [53,54,55,56]. These proteins are typically used to analyze overall UPS activity or to evaluate inhibitors of the 26S proteasome. However, these GFP-constructs may also have the potential to be used as tools to measure 26S proteasome stimulation in cells using small molecules. The most effective constructs for this purpose would be the GFPU construct, which is GFP fused to a sixteen amino acid CL1 degron, or the UbV-GFP fusion, which contains a mutated, non-cleavable ubiquitin fused to GFP [53,55]. The presence of the degron in GFPU enhances the recognition and degradation of this protein in cells. A similar phenomenon occurs with the UbV-GFP protein, as the presence of ubiquitin induces polyubiquitination and causes more efficient degradation. These proteins could be used in a kinetic fluorescence assay to monitor the decrease in GFP fluorescence over time. In the presence of a 26S proteasome stimulator, it would be expected that GFP signal would decrease at a faster rate. However, these proteins may be degraded too quickly by the non-stimulated 26S proteasome to be adequately used in this context, requiring the use of a more stable construct.

For a longer study that would involve cell treatment with a stimulator for several hours, a GFP fusion that is less susceptible to degradation would be required. GFP is naturally a very stable protein and is not an efficient substrate of the UPS [53]. Therefore, GFP mutants have been created that are not as stable as native GFP, yet still have a longer half-life than the proteins mentioned above. One such fusion would be Ub-L-GFP, which utilizes the N-end rule to prevent quick degradation of GFP, yet is not as stable as the native M-GFP.53 The N-end rule suggests that having an arginine or an amino acid residue that is readily acetylated as the N-terminal residue enhances the recognition and degradation of that protein [57]. By replacing the methionine with a leucine as the N-terminal residue, GFP becomes destabilized and is more readily degraded by the UPS. This fusion protein could be a useful tool for the analysis of a 26S proteasome stimulator to determine enhanced degradation of Ub-L-GFP when the stimulator is present.

### 4.2. Radioactively Labeled Proteasome Substrates

One of the ways in which researchers study the rate of protein degradation by the proteasome is to use a radioactively labeled amino acid, such as [H3] phenylalanine ([H3]-Phe) [58]. By exposing cells to [H3]-Phe, this amino acid becomes incorporated into proteins. Varying the length of exposure allows for the monitoring of short-lived or long-lived proteins. The labeled proteins will then be degraded by either the proteasome or the lysosome, producing short peptides that are further degraded by other proteases into individual amino acids. The cells are then incubated with a large excess of non-labeled Phe to prevent reincorporation of the labeled amino acids into newly synthesized proteins. This also encourages the excretion of the labeled amino acids into the medium, as the non-labeled Phe is taken into the cells. For the evaluation of short-lived proteins, cycloheximide is included in the medium supplemented with the non-labeled Phe to prevent protein synthesis and allow for immediate analysis without a long incubation time. Following incubation with the non-labeled amino acid, aliquots of the medium can be taken at different time periods. The medium should contain the degradation products of proteins that have been excreted from the cell. To ensure that only degradation products are present, trichloroacetic acid (TCA) is added to the medium to precipitate any proteins. This solution can then be centrifuged to separate the precipitated proteins from the degradation products, which will remain in the supernatant. Once the aliquots of the medium have been removed from the plate, the cells can be dissolved in a solution of NaOH. Both the supernatants containing the degradation products from various time periods and the cell lysate containing the labeled proteins are placed into separate scintillation vials. By measuring the amount of [H3]-Phe in each sample, one can determine the rate of total protein degradation. Performing an additional experiment of dosing with a proteasome or autophagy inhibitor allows for the calculation of how much protein degradation is due to each pathway.

This method of analyzing proteasome activity has not been applied to a small molecule proteasome stimulator. However, such an experiment would provide information other assays fail to deliver. The method described above provides a proteasomal rate of degradation of cellular proteins, which is precisely what is impacted by a small molecule stimulator. Therefore, this assay could provide information pertaining to how the rate of protein degradation by the proteasome is affected by a small molecule stimulator in cells. Unfortunately, there is no way to differentiate degradation by the 26S proteasome versus the 20S CP, as the majority of proteasome inhibitors directly target the 20S CP. KDT-11 and RA-190 are known inhibitors of Rpn13, which is a ubiquitin-receptor of the 19S RP [59,60]. These inhibitors could potentially be used as a control to identify how much protein degradation is ubiquitin-independent. In addition, the majority of the proteins degraded by the 20S CP are known to be short-lived proteins, such as regulatory and signaling proteins [6,61]. Performing this experiment with a 20S CP stimulator to monitor short-lived proteins could provide a difference in the rate of degradation of these proteins. However, further experimentation is needed to determine whether this method could be useful in analyzing a stimulator of the 20S CP.

### 4.3. In Vitro Fluorogenic and Bioluminescent Reporters

**Figure 5:**
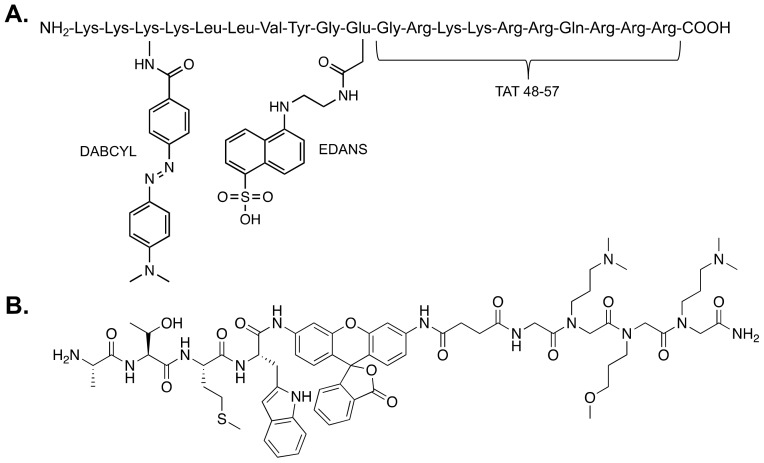
Structures of two fluorogenic reporters of proteasome activity that are effective in cells: (**A**) the Tat-Edans-Dabcyl (TED) peptide and (**B**) TBZ-1.

A few fluorogenic reporters have been developed to measure proteasome activity in cells, corresponding to the hydrolysis of the reporter [62,63]. One such reporter is referred to as the TED peptide, which stands for Tat-Edans-Dabcyl (Figure 5A) [62]. Ten amino acids from the transfer domain of the Tat protein were added to the C-terminus of this reporter to help with cell permeability [64]. The amino acid sequence between the FRET pair, Edans-Dabcyl, contains the LLVY cleavage-determining motif, with the intention of achieving specificity for the β-5 subunit of the 20S CP. Further lysine residues were added to the N-terminus of the peptide to ensure a net positive charge and promote membrane penetration. Analysis of this reporter revealed that the primary cleavage site is following the tyrosine; however, other cleavage products are also produced. Incubating the TED peptide with various cell lines produced an increase in fluorescence of Edans, associated with cleavage of the peptide by the proteasome. A maximum fluorescence signal was observed after 15 min of incubation.

Another reporter, TBZ1, published recently by Zerfas et al. is effective at selectively monitoring immunoproteasome activity in cells (Figure 5B) [63]. This reporter utilizes a rhodamine moiety for its source of fluorescence, following reporter hydrolysis, and a peptoid portion to increase cell permeability. Both TBZ1 and the TED peptide are able to monitor proteasome activity in cells in a kinetic fluorescence assay. Though these methods have not been applied to stimulators of any isoform of the proteasome, each offers an opportunity to directly monitor enhanced proteasomal activity in cells in the presence of a small molecule stimulator.

In addition to these fluorogenic reporters, a similar way of assessing proteasome activity utilizes bioluminescent reporters [65]. These reporters are structurally based on the AMC fluorogenic reporters often used in biochemical assays to monitor the individual activities of the proteasome. Instead of having an AMC group on the C-terminus, this group is substituted for aminoluciferin, which can be utilized by luciferase to produce light. The amount of light produced is directly proportional to proteasome activity. These luminescent substrates can aptly measure the individual proteasome activities in cells, offering a unique tool to assess proteasome stimulators.

## 5. Structural Evaluation of Small Molecule Stimulators of the Proteasome

### 5.1. AFM

Atomic force microscopy (AFM) has been proven an effective method of identifying the various states of the 20S CP [66,67]. The gate of the 20S CP is formed by the N-termini of several α-subunits. In solution, the gate is able to flux between the open- and closed-state, with the majority of the 20S CP in the closed-state. AFM is able to determine the percentage of 20S CP in the open-or closed-state. Several small molecule stimulators of the 20S CP are believed to stabilize the open-gate conformation, which would increase the percentage of 20S CP in this state. Using AFM, several studies have been able to confirm the conformational changes associated with the binding of a small molecule stimulator [35,43]. In particular, the small molecule TCH-165 discovered by Njomen et al. was determined to increase the percentage of open-state 20S CP from 28% ± 4% in the control sample to 59% ± 3% in the presence of 2 μM TCH-165 [35]. AFM has been shown to distinguish between these states of the 20S CP; however, the resolution using this technique is not high enough to determine specific structural elements. To look at amino acid structure and small molecule binding, a higher resolution technique, such as cryo-electron microscopy (cryo-EM), needs to be used.

### 5.2. Cryo-EM

**Figure 6:**
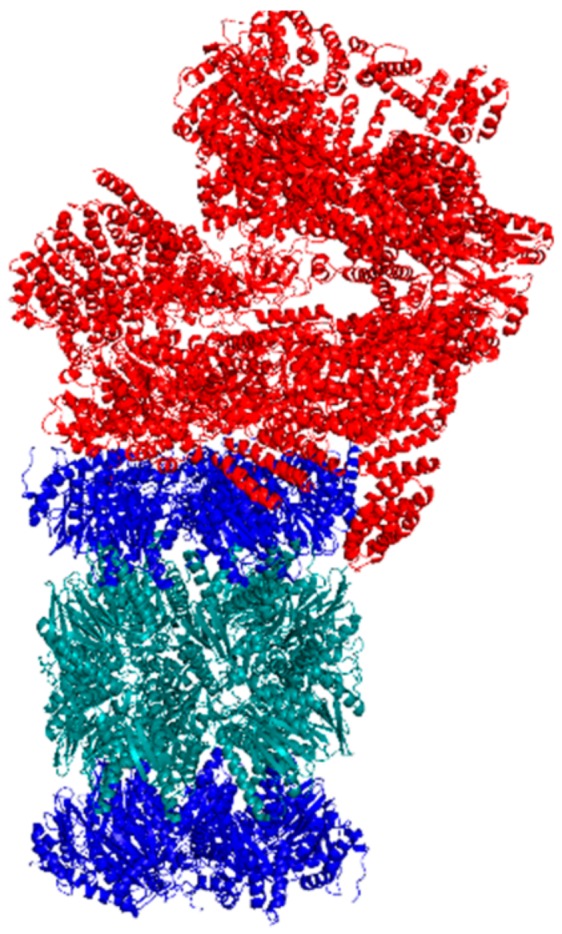
Cryo-EM structure of the human singly-capped 26S proteasome.

Cryo-EM has been successfully used to determine the structure of the human 20S CP and the 26S proteasome in different conformational states, Figure 6 [68,69]. Using this technique to analyze the binding site of a small molecule stimulator can illuminate the binding pocket and the key amino acids necessary for binding. Cryo-EM can also identify structural conformational changes that occur as a result of small molecule binding. This information is important to understand what subunits can be impacted to influence proteasomal activity. Identifying the binding pocket and key interactions can lead to structure-activity-relationship (SAR) studies that modify the structure of the small molecule to better interact with the proteasome.

## 6. Conclusions

The proteasome has been implicated as a potential target for stimulation in a number of disease states marked by protein accumulation. In this review, we discussed current methods of identifying and evaluating stimulators of various isoforms of the proteasome, such as high-throughput biochemical assays using fluorogenic substrates, cellular studies of GFP-fusion proteins, and structural experiments using AFM or cryo-EM. We also examined established methods of monitoring proteasome activity in cells that have the possibility of being used to analyze small molecule stimulators, which includes cellular fluorogenic or bioluminescent assays and using radio-labeled substrates to assess degradation by the proteasome.

Though the methods described here are effective at identifying stimulators of the proteasome, multiple assays are often required to (1) validate the small molecule as a proteasome stimulator, (2) determine the general mechanism of stimulation, and (3) assess the efficiency of the stimulator in cell models. The cellular assays used to this point are not high-throughput, requiring a significant amount of time and materials. It is, therefore, important to develop and apply more high-throughput cellular methods to identify and assess proteasome stimulators to directly evaluate their effectiveness in cell models. This would streamline the process of determining the therapeutic potential of a small molecule stimulator toward a protein accumulation disease.

## Figures and Tables

**Table 1:** Common fluorogenic peptide substrates used to assess proteasome activity.

Commonly Used Fluorogenic Peptide Substrates		
**Subunit specific cleavage**	β1	Z-LLE-NA Z-LLE-AMC Ac-nLPnLD-AMC
	β2	Boc-LRR-AMC Z-ARR-AMC Bz-VGR-AMC
	β5	Suc-LLVY-AMC Z-GGL-AMC Suc-AAF-AMC
Non-specific cleavage	FRET reporter	

## References

[1] Glick D., Barth S., Macleod K.F. (2010). Autophagy: Cellular and Molecular Mechanisms. J. Pathol..

[2] Sorokin A.V., Kim E.R., Ovchinnikov L.P. (2009). Proteasome System of Protein Degradation and Processing. Biochem. Biokhimiia.

[3] Ciechanover A. (1994). The Ubiquitin-Proteasome Proteolytic Pathway. Cell.

[4] Erales J., Coffino P. (2014). Ubiquitin-Independent Proteasomal Degradation. Biochim. Biophys. Acta BBA - Mol. Cell Res..

[5] Ehlinger A., Walters K.J. (2013). Structural Insights into Proteasome Activation by the 19S Regulatory Particle. Biochemistry.

[6] Ben-Nissan G., Sharon M. (2014). Regulating the 20S Proteasome Ubiquitin-Independent Degradation Pathway. Biomolecules.

[7] Ruggiano A., Foresti O., Carvalho P. (2014). ER-Associated Degradation: Protein Quality Control and Beyond. J. Cell Biol..

[8] Werner E.D., Brodsky J.L., McCracken A.A. (1996). Proteasome-Dependent Endoplasmic Reticulum-Associated Protein Degradation: An Unconventional Route to a Familiar Fate. Proc. Natl. Acad. Sci. USA.

[9] Hebert D.N., Molinari M. (2007). In and out of the ER: Protein Folding, Quality Control, Degradation, and Related Human Diseases. Physiol. Rev..

[10] Lindholm D., Wootz H., Korhonen L. (2006). ER Stress and Neurodegenerative Diseases. Cell Death Differ..

[11] Pickering A.M., Davies K.J.A. (2012). Degradation of Damaged Proteins—The Main Function of the 20S Proteasome. Prog. Mol. Biol. Transl. Sci..

[12] Asher G., Reuven N., Shaul Y. (2006). 20S Proteasomes and Protein Degradation “by Default”. BioEssays News Rev. Mol. Cell. Dev. Biol..

[13] Stadtmueller B.M., Hill C.P. (2011). Proteasome Activators. Mol. Cell.

[14] Fabre B., Lambour T., Garrigues L., Ducoux-Petit M., Amalric F., Monsarrat B., Burlet-Schiltz O., Bousquet-Dubouch M.-P. (2014). Label-Free Quantitative Proteomics Reveals the Dynamics of Proteasome Complexes Composition and Stoichiometry in a Wide Range of Human Cell Lines. J. Proteome Res..

[15] Heink S., Ludwig D., Kloetzel P.-M., Krüger E. (2005). IFN-Gamma-Induced Immune Adaptation of the Proteasome System Is an Accelerated and Transient Response. Proc. Natl. Acad. Sci. USA.

[16] Ferrington D.A., Gregerson D.S. (2012). Immunoproteasomes: Structure, Function, and Antigen Presentation. Prog. Mol. Biol. Transl. Sci..

[17] McNaught K.S.P., Belizaire R., Isacson O., Jenner P., Olanow C.W. (2003). Altered Proteasomal Function in Sporadic Parkinson’s Disease. Exp. Neurol..

[18] McNaught K.S., Jenner P. (2001). Proteasomal Function Is Impaired in Substantia Nigra in Parkinson’s Disease. Neurosci. Lett..

[19] Palmieri G., Cocca E., Gogliettino M., Valentino R., Ruvo M., Cristofano G., Angiolillo A., Balestrieri M., Rossi M., Di Costanzo A. (2017). Low Erythrocyte Levels of Proteasome and Acyl-Peptide Hydrolase (APEH) Activities in Alzheimer’s Disease: A Sign of Defective Proteostasis?. J. Alzheimers Dis..

[20] Gadhave K., Bolshette N., Ahire A., Pardeshi R., Thakur K., Trandafir C., Istrate A., Ahmed S., Lahkar M., Muresanu D.F. (2016). The Ubiquitin Proteasomal System: A Potential Target for the Management of Alzheimer’s Disease. J. Cell. Mol. Med..

[21] Arrasate M., Finkbeiner S. (2012). Protein Aggregates in Huntington’s Disease. Exp. Neurol..

[22] Kabashi E., Agar J.N., Strong M.J., Durham H.D. (2012). Impaired Proteasome Function in Sporadic Amyotrophic Lateral Sclerosis. Amyotroph. Lateral Scler. Off. Publ. World Fed. Neurol. Res. Group Mot. Neuron Dis..

[23] Hipkiss A.R. (2006). Accumulation of Altered Proteins and Ageing: Causes and Effects. Exp. Gerontol..

[24] Saez I., Vilchez D. (2014). The Mechanistic Links Between Proteasome Activity, Aging and Age-Related Diseases. Curr. Genomics.

[25] Liu Y., Liu X., Zhang T., Luna C., Liton P.B., Gonzalez P. (2007). Cytoprotective Effects of Proteasome Beta5 Subunit Overexpression in Lens Epithelial Cells. Mol. Vis..

[26] Chondrogianni N., Tzavelas C., Pemberton A.J., Nezis I.P., Rivett A.J., Gonos E.S. (2005). Overexpression of Proteasome Beta5 Assembled Subunit Increases the Amount of Proteasome and Confers Ameliorated Response to Oxidative Stress and Higher Survival Rates. J. Biol. Chem..

[27] Vilchez D., Saez I., Dillin A. (2014). The Role of Protein Clearance Mechanisms in Organismal Ageing and Age-Related Diseases. Nat. Commun..

[28] Chondrogianni N., Petropoulos I., Franceschi C., Friguet B., Gonos E.S. (2000). Fibroblast Cultures from Healthy Centenarians Have an Active Proteasome. Exp. Gerontol..

[29] Vilchez D., Boyer L., Morantte I., Lutz M., Merkwirth C., Joyce D., Spencer B., Page L., Masliah E., Berggren W.T. (2012). Increased Proteasome Activity in Human Embryonic Stem Cells Is Regulated by PSMD11. Nature.

[30] Lokireddy S., Kukushkin N.V., Goldberg A.L. (2015). CAMP-Induced Phosphorylation of 26S Proteasomes on Rpn6/PSMD11 Enhances Their Activity and the Degradation of Misfolded Proteins. Proc. Natl. Acad. Sci. USA.

[31] Leestemaker Y., de Jong A., Witting K.F., Penning R., Schuurman K., Rodenko B., Zaal E.A., van de Kooij B., Laufer S., Heck A.J.R. (2017). Proteasome Activation by Small Molecules. Cell Chem. Biol..

[32] Jones C.L., Njomen E., Sjögren B., Dexheimer T.S., Tepe J.J. (2017). Small Molecule Enhancement of 20S Proteasome Activity Targets Intrinsically Disordered Proteins. ACS Chem. Biol..

[33] Coleman R.A., Trader D.J. (2018). Development and Application of a Sensitive Peptide Reporter to Discover 20S Proteasome Stimulators. ACS Comb. Sci..

[34] Trader D.J., Simanski S., Dickson P., Kodadek T. (2017). Establishment of A Suite of Assays That Support the Discovery of Proteasome Stimulators. Biochim. Biophys. Acta.

[35] Njomen E., Osmulski P.A., Jones C.L., Gaczynska M., Tepe J.J. (2018). Small Molecule Modulation of Proteasome Assembly. Biochemistry.

[36] Gillette T.G., Kumar B., Thompson D., Slaughter C.A., DeMartino G.N. (2008). Differential Roles of the COOH Termini of AAA Subunits of PA700 (19 S Regulator) in Asymmetric Assembly and Activation of the 26 S Proteasome. J. Biol. Chem..

[37] Kisselev A.F., Kaganovich D., Goldberg A.L. (2002). Binding of Hydrophobic Peptides to Several Non-Catalytic Sites Promotes Peptide Hydrolysis by All Active Sites of 20 S Proteasomes EVIDENCE FOR PEPTIDE-INDUCED CHANNEL OPENING IN THE α-RINGS. J. Biol. Chem..

[38] Dal Vechio F.H., Cerqueira F., Augusto O., Lopes R., Demasi M. (2014). Peptides That Activate the 20S Proteasome by Gate Opening Increased Oxidized Protein Removal and Reduced Protein Aggregation. Free Radic. Biol. Med..

[39] Tanaka K., Yoshimura T., Ichihara A. (1989). Role of Substrate in Reversible Activation of Proteasomes (Multi-Protease Complexes) by Sodium Dodecyl Sulfate. J. Biochem. (Tokyo).

[40] Ruiz de Mena I., Mahillo E., Arribas J., Castaño J.G. (1993). Kinetic Mechanism of Activation by Cardiolipin (Diphosphatidylglycerol) of the Rat Liver Multicatalytic Proteinase. Biochem. J..

[41] Huang L., Ho P., Chen C.-H. (2007). Activation and Inhibition of Proteasomes by Betulinic Acid and Its Derivatives. FEBS Lett..

[42] Coleman R.A., Muli C.S., Zhao Y., Bhardwaj A., Newhouse T.R., Trader D.J. (2019). Analysis of Chain Length, Substitution Patterns, and Unsaturation of AM-404 Derivatives as 20S Proteasome Stimulators. Bioorg. Med. Chem. Lett..

[43] Giżyńska M., Witkowska J., Karpowicz P., Rostankowski R., Chocron E.S., Pickering A.M., Osmulski P., Gaczynska M., Jankowska E. (2019). Proline- and Arginine-Rich Peptides as Flexible Allosteric Modulators of Human Proteasome Activity. J. Med. Chem..

[44] Wilk S., Orlowski M. (1983). Evidence That Pituitary Cation-Sensitive Neutral Endopeptidase Is a Multicatalytic Protease Complex. J. Neurochem..

[45] Liggett A., Crawford L.J., Walker B., Morris T.C.M., Irvine A.E. (2010). Methods for Measuring Proteasome Activity: Current Limitations and Future Developments. Leuk. Res..

[46] Kisselev A.F., Goldberg A.L. (2005). Monitoring Activity and Inhibition of 26S Proteasomes with Fluorogenic Peptide Substrates. Methods in Enzymology.

[47] Schmidtke G., Holzhütter H.-G., Bogyo M., Kairies N., Groll M., de Giuli R., Emch S., Groettrup M. (1999). How an Inhibitor of the HIV-I Protease Modulates Proteasome Activity. J. Biol. Chem..

[48] Schmidtke G., Emch S., Groettrup M., Holzhutter H.G. (2000). Evidence for the Existence of a Non-Catalytic Modifier Site of Peptide Hydrolysis by the 20 S Proteasome. J. Biol. Chem..

[49] Schneider-Poetsch T., Ju J., Eyler D.E., Dang Y., Bhat S., Merrick W.C., Green R., Shen B., Liu J.O. (2010). Inhibition of Eukaryotic Translation Elongation by Cycloheximide and Lactimidomycin. Nat. Chem. Biol..

[50] Zhou P., Dickson R.C., Mendenhall M.D. (2004). Determining Protein Half-Lives. Signal Transduction Protocols.

[51] Kastrop P.M., Hulshof S.C., Bevers M.M., Destrée O.H., Kruip T.A. (1991). The Effects of Alpha-Amanitin and Cycloheximide on Nuclear Progression, Protein Synthesis, and Phosphorylation during Bovine Oocyte Maturation in Vitro. Mol. Reprod. Dev..

[52] Hochstrasser M. (1996). Ubiquitin-Dependent Protein Degradation. Annu. Rev. Genet..

[53] Dantuma N.P., Lindsten K., Glas R., Jellne M., Masucci M.G. (2000). Short-Lived Green Fluorescent Proteins for Quantifying Ubiquitin/Proteasome-Dependent Proteolysis in Living Cells. Nat. Biotechnol..

[54] Melvin A.T., Woss G.S., Park J.H., Waters M.L., Allbritton N.L. (2013). Measuring Activity in the Ubiquitin-Proteasome System: From Large Scale Discoveries to Single Cells Analysis. Cell Biochem. Biophys..

[55] Greussing R., Unterluggauer H., Koziel R., Maier A.B., Jansen-Dürr P. (2012). Monitoring of Ubiquitin-Proteasome Activity in Living Cells Using a Degron (Dgn)-Destabilized Green Fluorescent Protein (GFP)-Based Reporter Protein. J. Vis. Exp. JoVE.

[56] Menéndez-Benito V., Heessen S., Dantuma N.P. (2005). Monitoring of Ubiquitin-Dependent Proteolysis with Green Fluorescent Protein Substrates. Methods Enzymol..

[57] Varshavsky A. (2011). The N-End Rule Pathway and Regulation by Proteolysis. Protein Sci. Publ. Protein Soc..

[58] Sha Z., Zhao J., Goldberg A.L. (2018). Measuring the Overall Rate of Protein Breakdown in Cells and the Contributions of the Ubiquitin-Proteasome and Autophagy-Lysosomal Pathways. Methods Mol. Biol. Clifton NJ.

[59] Anchoori R.K., Karanam B., Peng S., Wang J.W., Jiang R., Tanno T., Orlowski R.Z., Matsui W., Zhao M., Rudek M.A. (2013). A Bis-Benzylidine Piperidone Targeting Proteasome Ubiquitin Receptor RPN13/ADRM1 as a Therapy for Cancer. Cancer Cell.

[60] Trader D.J., Simanski S., Kodadek T. (2015). A Reversible and Highly Selective Inhibitor of the Proteasomal Ubiquitin Receptor Rpn13 Is Toxic To Multiple Myeloma Cells. J. Am. Chem. Soc..

[61] Dyson H.J., Wright P.E. (2005). Intrinsically Unstructured Proteins and Their Functions. Nat. Rev. Mol. Cell Biol..

[62] Urru S.A.M., Veglianese P., De Luigi A., Fumagalli E., Erba E., Gonella Diaza R., Carrà A., Davoli E., Borsello T., Forloni G. (2010). A New Fluorogenic Peptide Determines Proteasome Activity in Single Cells. J. Med. Chem..

[63] Zerfas B.L., Trader D.J. (2019). Monitoring the Immunoproteasome in Live Cells Using an Activity-Based Peptide–Peptoid Hybrid Probe. J. Am. Chem. Soc..

[64] Järver P., Langel Ü. (2006). Cell-Penetrating Peptides—A Brief Introduction. Biochim. Biophys. Acta BBA - Biomembr..

[65] Moravec R.A., O’Brien M.A., Daily W.J., Scurria M.A., Bernad L., Riss T.L. (2009). Cell-Based Bioluminescent Assays for All Three Proteasome Activities in a Homogeneous Format. Anal. Biochem..

[66] Osmulski P.A., Gaczynska M. (2000). Atomic Force Microscopy Reveals Two Conformations of the 20 S Proteasome from Fission Yeast. J. Biol. Chem..

[67] Gaczynska M., Osmulski P.A. (2011). Atomic Force Microscopy of Proteasome Assemblies. Methods Mol. Biol. Clifton NJ.

[68] Huang X., Luan B., Wu J., Shi Y. (2016). An Atomic Structure of the Human 26S Proteasome. Nat. Struct. Mol. Biol..

[69] Dong Y., Zhang S., Wu Z., Li X., Wang W.L., Zhu Y., Stoilova-McPhie S., Lu Y., Finley D., Mao Y. (2019). Cryo-EM Structures and Dynamics of Substrate-Engaged Human 26S Proteasome. Nature.

